# Efficacy of GnRH-a in combination with dienogest therapy in endometriosis and its effects in recurrence and pregnancy status

**DOI:** 10.2478/abm-2026-0005

**Published:** 2026-04-30

**Authors:** Ke Zhou, Rong Zhu, Yue Jin

**Affiliations:** Department of Obstetrics and Gynecology, The First Affiliated Hospital, College of Medicine, Zhejiang University, Hangzhou 310006, China

**Keywords:** endometriosis, GnRH-a, inflammatory indicators, pregnancy, sex hormone levels

## Abstract

**Background:**

Endometriosis (EMT) is a common gynecologic and hormone-dependent disease that seriously affects patients' quality of life and currently faces certain challenges in clinical treatment.

**Objective:**

To observe the clinical efficacy of gonadotropin-releasing hormone agonists (GnRH-a) in combination with Dienogest treatment in EMT and its effects on recurrence and pregnancy.

**Methods:**

In a retrospective study, 254 patients with EMT from The First Affiliated Hospital, College of Medicine, Zhejiang University were selected between October 2020 and December 2021, and were divided into DT group (Dienogest treatment group) and GD group (GnRH-a combined with dienogest treatment group) according to the treatment modality, both groups were treated with Dienogest, and the GD group was additionally treated with GnRH-a. The primary assessment of both groups was clinical efficacy, recurrence rate and pregnancy (cumulative pregnancy rate, live birth rate, miscarriage rate, multiple pregnancy rate, and ectopic pregnancy rate). Secondary outcomes included the antibody indicator positive rate (anti-endometrial antibodies [AEmAb], anti-sperm antibodies [AsAb], and anti-zona pellucida antibodies [AZpAb]), sex hormone levels (luteinizing hormone [LH], follicle-stimulating hormone [FSH], and estradiol [E2]), serum inflammatory indicator levels (Interleukin-6 [IL-6], C-reactive protein [CRP], tumor necrosis factor-α [TNF-α]), visual analog scale (VAS) scores, and adverse reactions.

**Results:**

After treatment, all indicators improved in both groups (*P* < 0.05). The clinical efficacy, cumulative pregnancy rate, and live birth rate of patients in the GD group were markedly higher than those in the DT group (*P* < 0.05). The recurrence rate, positive rate of antibody indicator, sex hormone level, serum inflammation indicator, VAS score, and incidence of adverse reactions of patients in the GD group were remarkably below the DT group (*P* < 0.05). No marked discrepancies were found in the rates of miscarriage, multiple pregnancy, and ectopic pregnancy in both groups (*P* > 0.05).

**Conclusion:**

The efficacy of GnRH-a combined with Dienogest in the treatment of EMT is remarkable, which can effectively reduce the inflammatory response, sex hormone levels, and recurrence rate, and markedly improve pregnancy, which is worth promoting its use in the clinic.

Endometriosis (EMT) is a gynecologic disease that develops when endometrial cells (glands and mesenchyme), which are supposed to grow in the uterine cavity, appear through the fallopian tubes in parts of the uterine cavity other than the coated endometrium and the muscular layer of the uterus [1]. EMT is a gynecologic disease that occurs most often in women of childbearing age and is estrogen-dependent. The clinical manifestations of EMT are varied, and is broadly classified into ovarian, peritoneal, and deep-infiltrating types according to the location of its occurrence and depth of its infiltration [2]. Dysmenorrhea, chronic pelvic pain, and secondary infertility caused by EMT seriously affect women's quality of life and physical and mental health, and even more so, the normal life and stability of the family [3]. The most important lesion of EMT is adhesion, including pelvic adhesion, tubal obstruction, or peripheral adhesion, which leads to infertility by interfering with several aspects of ovulation, fertilization, and implantation. Cyclic bleeding occurs with menstrual changes after EMT, and cysts are formed locally in the ovary, and intracystic fluid leaks out when there is a fissure in the wall of the capsule, causing inflammatory reaction and fibrosis in the tissues around the ovary, normal stromal defects, abnormal function of granulosa cells of the affected follicle, and abnormal hormone levels, which leads to abnormal ovulation in the ovary [4]. EMT reduces the tolerance of the normal endometrium in the uterine cavity for fertilized eggs, resulting in intrauterine implantation disorders. Ectopic implantation lesions also increase the number and activity of macrophages, which mediate immune and inflammatory responses, leading to localized pelvic adhesions and fibrosis, thus causing impaired egg pickup at the umbilical end of the fallopian tube, affecting follicular development, inhibiting ovulation, and promoting corpus luteum lysis, leading to infertility [5]. EMT with infertility is one of the current challenges in clinical treatment in the field of obstetrics and gynecology and reproduction.

The fundamental aims of current treatment for EMT are to reduce and remove lesions, to treat and promote fertility, and to prevent and minimize recurrence. The main treatment modalities for EMT include expectant, pharmacologic, surgical, or combination therapy. Their treatment options vary depending on the severity of the disease, the patient's age, and fertility [6]. If EMT is severe, or if a pelvic examination reveals definite endometriotic nodules, pharmacologic or surgical treatment is necessary. Laparoscopic surgery for those lesions that cannot be completely removed will continue to proliferate, grow, and invade under the action of estrogen and progesterone, which can lead to postoperative recurrence and affect the outcome of surgery and postoperative conception [7]. Gonadotropin-releasing hormone (GnRH) is an endocrine hormone synthesized and secreted by hypothalamic nerve cells, which induces the hypothalamic pituitary gland to synthesize and release luteinizing hormone (LH), and reduces the levels of follicle-stimulating hormone (FSH), progesterone, and LH, thus exerting an inhibitory effect on the ovaries and decreasing the secretion of estrogen and progesterone [8]. Gonadotropin-releasing hormone agonists (GnRH-a) is an analog of natural GnRH, and the more clinically used ones are treprostinil acetate and leuprolide acetate [9]. Triprolidine is a synthetic analog of natural GnRH, a 10-peptide analog, and leuprolide is a 9-peptide analog of GnRH. The structure of the synthetic analog of this peptide has greatly increased binding to the receptor, producing competitive inhibition, competition for GnRH receptors, and faster onset of action, while the high degree of similarity gives it a natural GnRH-like effect and produces like-for-like effects [10]. GnRH-a inhibits the release of estrogen and progesterone and controls estrogen levels within the menopausal level, causing a temporary artificial menopausal state to delay and prevent recurrence. The effi-cacy of GnRH-a in relieving EMT symptoms and effectively delaying and inhibiting recurrence after laparoscopic resection has been widely reported [11].

Dienogest is a progestin containing both a progesterone derivative and 19-nortestosterone. The drug is a highly selective progesterone receptor agonist with moderate inhibitory effects on the hypothalamic-pituitary-ovarian gonadal axis, which was approved in China in 2018 and is now widely used [12]. The principle of Dienogest is to inhibit local estrogen production by down-regulating estrogen receptors and interfering with aromatase and 17β-hydroxysteroid dehydrogenase, as well as inhibiting cell proliferation and inflammatory response occurrence. However, the use of Dienogest alone has certain side effects, such as irregular vaginal bleeding, headache, depression, and water and sodium storage [13].

Currently, there are many knowledge gaps in the field of EMT treatment that need to be filled. Although Dienogest tablets have emerged as a promising agent in EMT treatment, their synergistic effect with GnRH-a, mechanism of action, and impact on patients' recurrence and pregnancy have not been fully and systematically investigated. With a keen focus on this, the present study is actively engaged in the exploration of this critical area and is dedicated to filling an important knowledge gap. In the course of in-depth observation of the clinical efficacy of GnRH-a combined with Dienogest tablets in the treatment of EMT patients, this study analyzed the recurrence of EMT after treatment and comprehensively tracked the pregnancy status of the patients, so as to explore the effect of the combined treatment in depth. Through an exhaustive compilation and analysis of complications and adverse reactions, this study clarifies for the first time the efficacy and safety of the combined use of the 2 treatments under specific samples and conditions. Previously, there have been studies on GnRH-a alone to regulate hormone levels and Dienogest tablets alone to inhibit ectopic endothelial growth, but the synergistic effect of the combination of the 2 in reducing the risk of recurrence and improving the outcome of pregnancy lacks sufficient data and in-depth analysis of the mechanism. The results of this study not only provide clinicians with more effective treatment options and help them to develop more optimal and personalized treatment plans for patients, but also further promote innovative breakthroughs and improvements in EMT treatment strategies, which will bring new hope and improve the quality of life for patients suffering from EMT.

## Methods

The study was performed in compliance with the Declaration of Helsinki. It was approved by ethical committees of the First Affiliated Hospital, College of Medicine, Zhejiang University ([2025B]IIT Ethics Approval No. 0730). The written informed consent was obtained from the patients.

**Figure 1.**
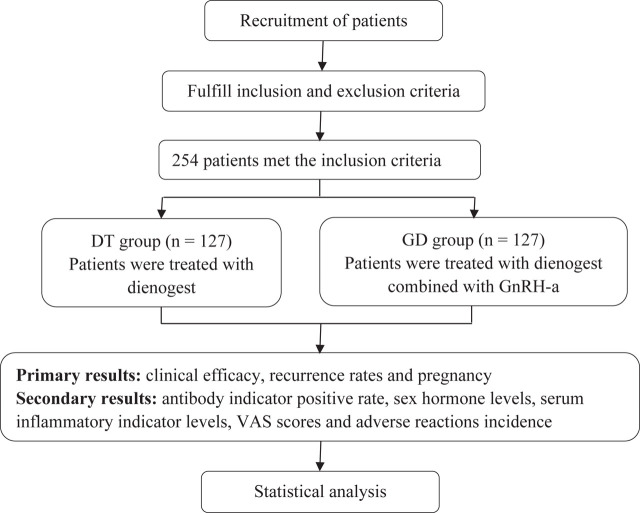
Flow chart of study. VAS, visual analog scale.

This study is a systematic evaluation and integration aiming to comparatively analyze the clinical efficacy of GnRH-a in combination with Dienogest tablets treatment in EMT patients, and to further assess its impact on patients' recurrence and pregnancy. This study was a retrospective study, and 254 EMT patients admitted from The First Affiliated Hospital, College of Medicine, Zhejiang University from October 2020 to December 2021 were selected, and categorized into both groups based on the different interventions, the DT group (Dienogest treatment group) and the GD group (GnRH-a combined with dienogest treatment group). The flow chart of this study is shown in **Figure 1**.

### Inclusion and exclusion criteria

Inclusion criteria: (1) Meet the clinical diagnostic criteria for ovarian EMT [14], and after admission to the hospital, after pathological examination, it was determined that there were no complications and that the lesions were benign; (2) The patients had not taken hormone-containing drugs within 6 months of the treatment; (3) Aged 21–56 years; (4) Patients without other gynecological diseases; (5) The patients had a good degree of adherence, and they were willing to cooperate with the treatment plan formulated by the study; (6) Patients were in good overall mental state, basically healthy, and could truthfully express their complaints about the symptoms and answer relevant questions from the medical staff; (7) Those who could tolerate the drugs involved in this study; (8) The patients and their family members were informed and agreed, and signed an informed consent form.

Exclusion criteria: (1) Any site or type of malignant tumor, such as gynecological malignant tumors; (2) Combined with hemorrhagic coagulation dysfunction, or patients with serious liver or renal function defects, serious cardiovascular disease or other more serious gynecological endocrine diseases; (3) Combined with chronic infectious diseases; (4) Pregnant or lactating patients; (5) Patients who have been involved in clinical drug trials or clinical studies; (6) Patients with combined neurological or psychiatric diseases that make it difficult for them to communicate normally; (7) Patients who request to stop treatment or are automatically discharged from the hospital for personal reasons; (8) Patients with other infertility factors; (9) Psychiatric illnesses; (10) Patients who are allergic to the medication used in this study; (11) Other conditions that the study physicians think should not be included; (12) Other conditions that affect the indicators of subsequent observation.

### Intervention

Patients in both groups were treated with Dienogest tablets, and patients were orally administered Dienogest on the first day of menstruation (Nanjing Bai Jingyu Pharmaceutical Co., Ltd., State Pharmaceutical License: H20213439, specification: 2 mg/tablet), once a day, 1 tablet each time, for 6 consecutive months. In the course of drug administration, closely observe the patient's adverse reactions, such as irregular vaginal bleeding, breast distension and pain, etc., and give appropriate treatment and guidance in time. At the same time, patients were told to maintain a routine and to avoid missing the medication, and if there was a missed dose, it should be made up as soon as possible when remembered. However, if it is close to the time of the next dose, there is no need to make up for the missed dose, and the next dose should be taken at the normal time.

Patients in the GD group were additionally treated with GnRH-a, and leuprolide acetate microspheres for injection on the first day of menstruation (State Pharmaceutical License No. J20150109, 3.75 mg) were injected subcutaneously with 3.75 mg/times, and then every 28 d for a total of 6 months of treatment. During the treatment period, as GnRH-a may lead to hypoestrogenic symptoms, such as hot flashes, night sweats, bone loss, etc., closely monitor the patient's symptomatic changes, and give calcium supplementation and lifestyle guidance if necessary, e.g., encouraging the patient to appropriately increase outdoor activities and sunbathing, to promote the absorption of calcium.

### Nursing programme (The nursing programme was the same for both groups)

Pain management: both groups paid close attention to pain symptoms during treatment. For patients with mild pain, they were instructed to relieve pain through hot compresses on the lower abdomen (avoiding wounds), appropriate rest, meditation, listening to music, etc. For patients with moderate-to-severe pain, they were given non-steroidal anti-inflammatory drugs, such as ibuprofen, according to the doctor's advice, and the medication regimen was adjusted if necessary.

Diet and lifestyle guidance: All patients received diet and lifestyle advice. In terms of diet, patients were encouraged to consume foods rich in protein, vitamins, and minerals, such as lean meat, fish, fresh vegetables, and fruits, in order to strengthen the body's resistance. Meanwhile, spicy and stimulating foods as well as caffeine- and alcohol-containing beverages should be avoided to reduce the adverse stimulation to the body. In terms of lifestyle, patients are advised to maintain a regular work and rest schedule, ensure sufficient sleep, and sleep for 7–8 h a day. Avoid strenuous exercise, heavy labor, and sexual life for 1 month after surgery. After 1 month, moderate exercise such as walking and yoga can be resumed gradually, but movements that increase abdominal pressure, such as abdominal rolls and deep squats, should be avoided.

Psychological support: considering that patients with EMT are prone to anxiety, depression, and other adverse emotions due to long-term suffering from the disease, which affects the therapeutic effect and the quality of life, psychological support is provided to both groups of patients in the course of treatment. Through adequate communication with the patients to understand their psychological state, give comfort and encouragement, and help patients establish confidence in overcoming the disease. If necessary, patients are advised to seek help from professional psychological counselors.

Regular follow-up: Both groups of patients need regular follow-up during and after the treatment. If symptoms such as worsening of dysmenorrhea, abnormal vaginal bleeding, or infertility worsen during the follow-up period, the patients should consult the doctor in time. During the treatment period, as GnRH-a may lead to hypoestrogenic symptoms, such as hot flashes, night sweats, bone loss, etc., patients should be closely monitored for symptomatic changes, and calcium supplementation and lifestyle guidance should be given when necessary, such as encouraging patients to increase outdoor activities and sunbathing more often to promote calcium absorption.

#### Observation indicators

##### Clinical efficacy

Compare the clinical efficacy of the 2 groups. Efficacy evaluation criteria: after completing the treatment, the symptoms of abnormal menstruation, dysmenorrhea and lower abdominal pain basically disappeared, and the ultrasound showed that there were no cysts in the pelvis was considered as obvious effect; the symptoms of abnormal menstruation, dysmenorrhea and lower abdominal pain were significantly relieved, and the ultrasound showed that there were no cysts in the pelvis was considered as effective; the symptoms of abnormal menstruation, dysmenorrhea and lower abdominal pain were not relieved or aggravated, and the ultrasound showed that there were cysts in the pelvis was considered as ineffective. Total effective rate = (obvious effect + effective)/total × 100%.

##### Recurrence rate

Record the recurrence of patients in both groups during the follow-up period.

##### Pregnancy

Record the rate of cumulative pregnancy, live birth, miscarriage, multiple pregnancy, and ectopic pregnancy of both groups during the follow-up period. The presence of assisted reproductive technologies to assist conception in both groups was 3.94% (5/127) in the DT group, and 3.15% (4/127) in the GD group, and no significant difference was observed when comparing both groups (*P* > 0.05).

##### Antibody indicator positive rate

Fasting venous blood of 3 mL was drawn from patients, centrifuged, and kept refrigerated for testing, and an enzyme-linked immunosorbent assay was used to determine and calculate the positivity rates of anti-endometrial antibody (AEmAb), anti-sperm antibody (AsAb), and anti-zona pellucida antibody (AZpAb) in peripheral blood serum samples of patients before and after the interventions [15].

The kits used were human AEMAb ELISA test kit (Item No.: CB13562-Hu, Shanghai COIBO Biotechnology Co., Ltd.), human AsAb ELISA test kit (Item No.: CB11843-Hu, Shanghai COIBO Biotechnology Co., Ltd.), and human AZPA ELISA test kit (Item No.: CB13207-Hu, Shanghai COIBO Biotechnology Co., Ltd.).

##### Sex hormone levels

Before and after treatment, on the 5th to 7th day of the menstrual cycle, 5 mL of fasting peripheral blood was collected from both groups, and the supernatant was retained for measurement. Serum FSH, LH, and estradiol (E2) levels were measured by radioimmunoassay [16].

##### Serum inflammatory indicators

Referring to the research method of Kanlioglu et al. [17], 3 mL of fasting venous blood was drawn from patients, centrifuged and processed, and then preserved in cold storage to be detected, and the levels of C-reactive protein (CRP), interleukin-6 (IL-6), and tumor necrosis factor-α (TNF-α) were measured using enzyme-linked immunosorbent assay.

The kits used were human IL-6 ELISA kit (Item No.: ml027379, Shanghai Enzyme-linked Biotechnology Co., Ltd.), human CRP ELISA kit (Item No.: ml057570, Shanghai Enzyme-linked Biotechnology Co., Ltd.), and human TNF-α ELISA kit (Item No.: ml077385, Shanghai Enzyme-linked Biotechnology Co., Ltd.).

##### VAS score

The level of pain before and after treatment (1 month after the end of the treatment cycle) was compared in both groups. The visual analog scale (VAS) was used to assess before and after treatment, respectively [18].

##### Adverse reactions

The occurrence of adverse reactions during treatment was recorded in both groups, including nausea/vomiting, hot flashes/sweating, headache, vaginal bleeding, pelvic inflammatory disease, ovarian hyperstimulation syndrome, osteoporosis, and so on.

#### Follow-up visits

Follow-up visits at 24 months post-treatment were primarily scheduled in this study to assess the durability of the effects and to address any potential adverse reactions or problems.

Sample size was based on a power analysis performed with G*Power 3.1.9.7 computer software to determine the sample size required to detect a statistically significant difference. The sample size was calculated based on the primary outcome of clinical efficacy. Considering an α level of 0.05 and 90% efficacy, we calculated that a sample size of 108 patients was required for each group [19]. Considering the potential uncertainties, the sample sizes selected for this study were the DT group (n = 127) and the GD group (n = 127), and we believe that the sample sizes in this study are able to draw reliable conclusions.

#### Statistical methods

SPSS27.0 statistics software was applied for analysis of the data. Measurements that conform to normally distributed value are represented as (*x* s), and comparisons among groups adopts act pattern *t* examination independently, and counting data is expressed as a rate (%) using *x*^2^ test, with *P* < 0.05 indicating a statistical significance of the difference.

## Results

In this study, 254 patients with EMT diagnosed by surgical pathology or imaging were enrolled between October 2020 and December 2021 from The First Affiliated Hospital, College of Medicine, Zhejiang University. They were assigned to the DT group (n = 127) and GD group (n = 1 27) according to different interventions. The baseline demographic characteristics and baseline characteristics of the patients in both groups are shown in **Table 1**. Test analysis of the baseline demographic characteristics and clinical characteristics of the patients in both groups showed that there was no statistically significant difference between the groups (*P* > 0.05). This result indicates that the grouping effectively balanced the confounding factors affecting the efficacy of the treatment, ensured that both groups were homogeneous and comparable before treatment, and provided a reliable research basis for the subsequent analysis of the impact of combined treatment on the recurrence and pregnancy rates.

**Table 1.** Patient demographics and baseline disease characteristics

**Parameter**	**DT group (n = 127)**	**GD group (n = 127)**	***t/x* ^2^**	***P***	**Effect size**
Age (years)	33.13 ± 7.46	34.78 ± 8.53	1.641	0.102	−0.206
Infertility time (years)	24.59 ± 8.58	24.51 ± 8.43	−0.075	0.940	0.009
Height (years)	159.60 ± 5.20	158.67 ± 5.15	−1.432	0.153	0.180
Weight (kg)	64.75 ± 7.72	64.33 ± 7.06	−0.452	0.651	0.057
Body mass index (kg/m^2^)	23.42 ± 2.95	22.99 ± 2.52	−1.249	0.213	0.157
Ovarian cysts (single/double)	89/38	83/44	0.648	0.421	
Smoking (yes/no)	10/117	9/118	0.057	0.811	
Alcohol consumption (yes/no)	15/112	13/114	0.161	0.689	
Hypertension (yes/no)	23/104	25/102	0.103	0.749	
Diabetes (yes/no)	26/101	27/100	0.024	0.877	
Autoimmune disease (yes/no)	5/122	4/123	0.115	0.734	
Uterine fibroid tumor (yes/no)	40/87	38/89	0.074	0.786	
Cesarean section (yes/no)	48/79	46/81	0.068	0.795	
History of gynecologic surgery (yes/no)	10/117	11/116	0.052	0.820	
Temperature (°C)	36.37 ± 0.29	36.42 ± 0.30	1.350	0.178	0.169
Breathing (breaths/min)	17.05 ± 1.80	17.12 ± 1.85	0.306	0.760	−0.038
Heart rate (beat/min)	75.34 ± 7.15	74.79 ± 7.17	−0.612	0.541	0.077
Systolic blood pressure (mmHg)	119.52 ± 6.25	119.26 ± 6.14	−0.334	0.738	0.042
Diastolic blood pressure (mmHg)	75.91 ± 6.18	76.31 ± 5.75	0.534	0.594	−0.067

### Clinical efficacy

Combining the drug treatment effect of both groups, we analyzed clinical efficacy, and the results are presented in **Table 2**. The total effective rate of DT group patients was 80.31% (102/127), and GD group patients was 92.91% (118/127), which was statistically significant compared among the groups (*P* < 0.05). The results indicating better efficacy in GD group patients suggest that the clinical efficacy of GnRH-a combined with Dienogest in treating EMT patients is superior to Dienogest alone.

**Table 2.** Clinical efficacy analysis

**Group**	**Obvious effect (n)**	**Effective (n)**	**Ineffective (n)**	**Total effective rate n (%)**
DT group	50	52	25	102 (80.31)
GD group	58	60	9	118 (92.91)
*x* ^2^	7.236			
*P*	<0.05			

### Recurrence rate

We recorded the recurrence of patients in both groups during the follow-up period, and the recurrence rate of patients in the DT group was 12.60% (16/127) significantly higher than that of the GD group, which was 3.94% (5/127), with a statistically significant difference (*P* < 0.05). It shows that the combined treatment method used in this study can effectively reduce the recurrence rate of patients after treatment.

### Pregnancy

The pregnancy situation of patients in both groups is presented in **Table 3**, the cumulative pregnancy rate and live birth rate of patients in the DT group were 20.47% and 17.32%, respectively, which were obviously below GD group's 37.80% and 33.07% (*P* < 0.05). No obvious discrepancy in the comparison of rate of miscarriage, multiple pregnancy and ectopic pregnancy in both groups, indicating that the combined treatment method can effectively improve the cumulative pregnancy rate and live birth rate of the patients.

**Table 3.** Pregnancy

**Group**	**Cumulative pregnancy rate**	**Live birth rate**	**Miscarriage rate**	**Multiple pregnancy rate**	**Ectopic pregnancy rate**
DT group	26 (20.47)	22 (17.32)	4 (3.15)	0 (0.00)	2 (1.57)
GD group	48 (37.80)	42 (33.07)	2 (1.57)	0 (0.00)	0 (0.00)
*x* ^2^	7.868	6.827	0.205	-	2.020
*P*	<0.05	<0.05	0.651	-	0.155

### Antibody indicator positive rate

The results of the comparison of the antibody indicators' positivity rate of both groups of patients are displayed in **Table 4**. Before treatment, no obvious discrepancy in the positivity rates of AEmAb, AsAb, and AZpAb in both groups of patients. After treatment, the antibody indicator positive rates of patients in GD group were 7.09%, 6.30%, and 7.09%, which were obviously below the 17.32%, 17.32%, and 18.11% of the DT group (*P* < 0.05). This indicates that the combined treatment method can obviously improve the antibody indicator positive rate of the patients.

**Table 4.** Antibody indicators positive rate

**Norm**	**Time**	**DT group**	**GD group**	***x* ^2^**	***P***
AEmAb	Pre-treatment	38 (29.92)	37 (29.13)	0.024	0.877
	Post-treatment	22 (17.32)^*^	9 (7.09)^*^	4.735	<0.05
AsAb	Pre-treatment	39 (30.71)	40 (31.50)	0.011	0.917
	Post-treatment	22 (17.32)^*^	8 (6.30)^*^	5.944	<0.05
AZpAb	Pre-treatment	41 (32.28)	42 (33.07)	0.023	0.880
	Post-treatment	23 (18.11)^*^	9 (7.09)^*^	5.531	<0.05

* Discrepancy compared with pre-treatment, *P* < 0.05.

AEmAb, anti-endometrial antibody; AsAb, anti-sperm antibody; AZpAb, anti-zona pellucida antibody.

### Sex hormone levels

The results of the comparison of the sex hormone levels of both groups of patients are presented in **Table 5**. Before treatment, no obvious discrepancy in the comparison of sex hormone levels in both groups of patients (*P* > 0.05). After treatment, the FSH, LH, and E2 levels in DT group were 4.03 ± 1.22 U/L, 5.46 ± 1.21 U/L and 61.83 ± 12.91 pg/mL, respectively. The GD group were 3.22 ± 1.39 U/L, 4.21 ± 1.03 U/L and 50.22 ± 10.06 pg/mL, respectively, which were obviously decreased comparing with pre-treatment. GD group was markedly below DT group (*P* < 0.05). It indicates that the sex hormone levels of patients in both groups were markedly reduced after treatment, and the GD group had a better situation of marked improvement in sex hormone levels.

**Table 5.** Comparison of sex hormone levels (*x* ± s)

**Norm**	**Time**	**DT group**	**GD group**	***t***	***P***	**Effect size**
FSH (U/L)	Pre-treatment	5.78 ± 1.34	5.84 ± 1.56	0.329	0.743	−0.041
	Post-treatment	4.03 ± 1.22^*^	3.22 ± 1.39^*^	−4.936	<0.001	0.619
LH (U/L)	Pre-treatment	6.42 ± 1.46	6.51 ± 1.38	0.505	0.614	−0.063
	Post-treatment	5.46 ± 1.21^*^	4.21 ± 1.03^*^	−8.865	<0.001	1.112
E2 (pg/mL)	Pre-treatment	72.82 ± 13.37	72.52 ± 15.96	−0.162	0.871	0.020
	Post-treatment	61.83 ± 12.91^*^	50.22 ± 10.06^*^	−7.994	<0.001	1.003

* Discrepancy compared with pre-treatment, *P* < 0.05.

E2, estradiol; FSH, follicle-stimulating hormone; LH, luteinizing hormone.

### Inflammatory indicators

The results of the comparison of inflammatory indicators of patients in both groups are illustrated in **Table 6**. Before treatment, no remarkable discrepancy in the levels of CRP, TNF-α and IL-6 in both groups of patients when compared among the groups (*P* > 0.05). Post-treatment, the inflammatory indicator levels in the DT group were 4.03 ± 0.39 ng/L, 2.39 ± 0.30 ng/L, and 12.39 ± 1.66 ng/L, respectively, and the GD group were 3.25 ± 0.38 ng/L, 1.61 ± 0.29 ng/L, and 6.58 ± 1.67 ng/L, respectively, which were remarkably decreased versus pre-treatment. The GD group was markedly below the DT group (*P* < 0.05). It indicates that the inflammatory indicator levels were markedly reduced in both groups post-treatment, and patients in the GD group showed better improvement.

**Table 6.** Comparison of inflammatory indicators (*x* ± s, ng/L)

**Norm**	**Time**	**DT group**	**GD group**	***t***	***P***	**Effect size**
CRP	Pre-treatment	6.20 ± 0.38	6.21 ± 0.39	0.207	0.836	−0.026
	Post-treatment	4.03 ± 0.39^*^	3.25 ± 0.38^*^	−16.143	<0.001	2.026
TNF-α	Pre-treatment	5.07 ± 0.50	5.01 ± 0.48	−0.976	0.330	0.122
	Post-treatment	2.39 ± 0.30^*^	1.61 ± 0.29^*^	−21.067	<0.001	2.644
IL-6	Pre-treatment	16.06 ± 2.21	15.78 ± 2.70	−0.904	0.367	0.113
	Post-treatment	12.39 ± 1.66^*^	6.58 ± 1.67^*^	−27.807	<0.001	3.489

* Discrepancy compared with pre-treatment, *P* < 0.05.

CRP, C-reactive protein; IL-6, interleukin-6; TNF-α, tumor necrosis factor-α.

### VAS score

The VAS scores of both groups are demonstrated in **Table 7**. Before treatment, no marked discrepancy among the scores of both groups compared to each other (*P* > 0.05). Post-treatment, the score of patients in the DT group was 9.47 ± 1.50 scores, and the score of the GD group was 7.58 ± 1.38 scores, and the GD group was remarkably below the DT group (*P* < 0.05). It indicated that the pain level of both groups was relieved after treatment, and the patients in GD group had better pain relief.

**Table 7.** VAS score (*x* ± s, score)

	**DT group**	**GD group**	***t***	***P***	**Effect size**
Pre-treatment	13.18 ± 1.94	13.10 ± 2.18	−0.309	0.758	0.039
Post-treatment	9.47 ± 1.50	7.58 ± 1.38	−10.450	<0.001	1.311
*t*	−17.049	−24.111			
*P*	<0.001	<0.001			
Effect size	2.140	3.026			

VAS, visual analog scale.

### Adverse reactions

We followed up with the patients to observe the adverse reactions. Adverse reactions such as nausea/vomiting with varying degrees of severity occurred during the treatment period in both groups, as presented in **Table 8**. No remarkable discrepancy in the comparison of adverse reactions such as nausea/vomiting in both groups (*P* > 0.05). The total incidence of adverse reactions in patients in the DT group was 14.96% (19/127), which was remarkably above that in the patients in the GD group, which was 5.51% (7/127) (*P* < 0.05). This indicated that the therapeutic efficacy of the treatment used in the patients in the GD group was better, and the safety was higher.

**Table 8.** Occurrence of adverse reactions (n, %)

	**DT group**	**GD group**	***x* ^2^**	***P***
Nausea/vomiting	4 (3.15)	3 (2.36)	0.205	0.651
Hot flashes/sweating	1 (0.79)	0 (0.00)	1.005	0.316
Headache	2 (1.57)	1 (0.79)	0.338	0.561
Diarrhea	4 (3.15)	1 (0.79)	1.020	0.312
Vaginal bleeding	2 (1.57)	0 (0.00)	2.020	0.155
Pelvic inflammatory disease	2 (1.57)	0 (0.00)	2.020	0.155
Ovarian hyperstimulation syndrome	1 (0.79)	2 (1.57)	0.338	0.561
Urinary tract infection	2 (1.57)	0 (0.00)	2.020	0.155
Osteoporosis	1 (0.79)	0 (0.00)	1.005	0.316
Total response incidence	19 (14.96)	7 (5.51)	4.310	<0.05

## Discussion

EMT is a relatively common gynecological condition characterized histologically by the appearance of endometrial glandular bodies and stroma outside the uterine cavities, growth, infiltration, recurrent hemorrhage and formation of nodules or masses, which can cause extensive and severe adhesions [20]. According to relevant reports endometriotic lesions can be present in the bladder, thoracic and abdominal cavities, gas-trointestinal tract, urinary tract, cesarean section scars, nasal passages, and even on the skin surface, with the most common locations being the ovaries, Douglas' fossa, and uterosacral ligaments. In recent years, scholars have proposed that EMT should be regarded as a chronic disease similar to hypertension, heart disease, diabetes, and other chronic diseases, and that a chronic disease management diagnostic and therapeutic mindset should be developed, with a treatment concept of long-term or lifelong management [21]. Symptoms of EMT include dysmenorrhea, pain, infertility, or pelvic mass, which may have a certain impact on fertility, interpersonal relationships, and quality of life, and in severe cases, may further lead to deep infiltrative pelvic EMT, pelvic adhesions, and closure of the Douglas fossa, etc. [22].

Current treatments for EMT are surgical and pharmacologic [23]. Surgical treatment reduces recurrence rates, increases spontaneous pregnancy rates, and reduces pelvic pain. EMT is prone to recurrence due to the regeneration of residual lesions or formation of new lesions, and repeated surgeries may lead to further impairment of ovarian function, especially after reoperation for recurrent disease, which may lead to secondary damage [24]. Pharmacologic therapy is mainly based on GnRH-a and progestin analogs, including GnRH-α, Dienogest, and Chinese herbal medicine [25]. GnRH-a, which is characterized by a single dose with a long duration and few adverse effects, can improve patients' local symptoms by regulating their sex hormone levels, can assist in the process of rebalancing sex hormone levels in postoperative patients, and reduce the recurrence of ovarian EMT [26]. Dienogest, a 19-nortestosterone derivative, is a fourth-generation orally active progestin with high specificity for the progesterone receptor, and as a progestogen analog, it can achieve therapeutic goals by inhibiting the synthesis and secretion of estrogen and decreasing the endometrium's sensitivity to estrogen [27]. Therefore, the combination of GnRH-a and Dienogest was chosen for the treatment of EMT in this study to observe the clinical efficacy.

The results of this study demonstrated that after treatment, the indicators of both groups of patients were stati-stically significant compared with the pre-treatment. The clinical efficacy of the patients in the GD group, the cumulative pregnancy rate and live birth rate were remarkably above the DT group, and the recurrence rate was remarkably below the DT group (*P* < 0.05), and no remarkable discrepancy in the miscarriage rate, the rate of multiple pregnancies and the rate of ectopic pregnancies of the both groups of patients (*P* > 0.05). The reason was analyzed as GnRH-a is a GnRH, which can inhibit the proliferation and growth of endothelial cells by regulating the secretory effect of the pituitary gland, inhibit or reduce the size of the lesions, play the role of pharmacological resection of the lesions, and completely remove the tiny lesions and destroy the residual lesions with more serious adhesions [28]. The combination of GnRH-a and dinogestrel exerts a synergistic effect. From the perspective of the hormone regulation network, GnRH-a mainly acts at the level of the pituitary gland and strongly inhibits the secretion of gonadotropins, while Dinogestrel regulates hormone levels at multiple levels from the hypothalamus and ovaries. The combination of the 2 results in an all-round suppression of estrogen production and action, which more effectively inhibits endothelial growth, helping to restore a normal menstrual cycle and creating a favorable endocrine environment for pregnancy. This is consistent with the findings of the systematic review and network meta-analysis by Hodgson et al. [29].

AsAb, AEmAb, and AZpAb are a class of autoimmune antibodies capable of interfering with conception and causing miscarriage. Patients with multiple causes of infertility have a remarkably increased rate of antibody positivity in their serum compared to healthy women [30]. FSH, LH, and E2, as important sex hormones, show specific changes in patients with EMT. E2 is produced by ovarian follicles, corpus luteum, and placenta during pregnancy. When endometrial cells grow outside the uterus, estrogen is produced, resulting in increased levels of E2, and high levels of E2 can further stimulate follicular development and growth, leading to a corresponding increase in the concentration of FSH and LH [31]. FSH and LH are mainly secreted by the anterior pituitary gland, which plays a key role in regulating the female reproductive cycle. EMT patients, due to the impact of the disease on ovarian function, will experience abnormalities in the secretion and regulation of FSH and LH, which will further affect the ovarian ovulatory function and hormone levels, thus affecting their reproductive health [32]. The results demonstrated that the positive rate of antibody indicators, sex hormone levels, inflammation indicators, VAS scores, and the incidence of adverse reactions in patients in the GD group were remarkably below the DT group post-treatment (*P* < 0.05). The reason was analyzed as GnRH-a inhibits the release of gonadotropins from the anterior pituitary gland in order to reduce the estrogen level, promote the patients' autoantibodies to turn negative, and inhibit the aggregation of monocytes and neutrophils, which further reduces the inflammatory damage induced by the lesions [33]. Denogestrel also has anti-inflammatory properties, which can inhibit the expression and release of inflammatory factors and reduce the local inflammatory reaction of ectopic endometrial tissue. The combination of the 2 complements each other in terms of immune regulation and inflammation inhibition, improving the immune microenvironment of the patient's body, lowering the levels of FSH, LH and E2, reducing the adverse effects of inflammation on the reproductive system, relieving symptoms such as dysmenorrhea, and thus improving the effect of ovarian reserve function [34]. Han et al. [35] reported similar findings in a study of the effects of Psychological Intervention Combined with GnRH-a on patients after ovarian EMT laparoscopy. These results indicate that the combination of the 2 can effectively improve the positive rate of antibody indexes and sex hormone levels in patients, reduce the inflammatory response, and have a low recurrence rate and a high degree of safety, which also provides more scientific basis and therapeutic strategies for clinical treatment.

This study has some limitations. The sample size was relatively small and failed to cover all the different conditions of EMT, which may lead to bias in the study results and affect the extrapolation and reliability of the conclusions. A limitation of the single-center study was that there were differences in the patients' own underlying conditions, such as recurrence rates, pregnancy outcomes and other indicators, which were only recorded as occurring or not and not analyzed in more detail, which may affect the generalizability of the results. In terms of research design, this study used retrospective data collection. In retrospective studies, there are certain inherent flaws in data collection. Some patients had missing medical records, which made it impossible to accurately assess the size and change in location of their ectopic lesions before treatment, etc. This may affect the accuracy of the assessment of disease progression and treatment effects. In terms of data collection, as this study was a retrospective collection of data over multiple years, there were inconsistencies in the standards and methods of data collection in different periods. For the testing of inflammatory indicators, the testing instruments and methods were updated over time, and the comparability of test results from different batches was reduced. This inconsistency in data collection may lead to variable data quality and affect the reliability of the study results. In addition, the relatively short follow-up period did not allow for adequate assessment of the long-term effects and safety of the treatment. Therefore, the sample size and follow-up period should be further expanded in future studies to more extensively evaluate the efficacy and safety of GnRH-a in combination with Dienogest treatment in EMT patients.

## Conclusion

This study analyzed the clinical efficacy of GnRH-a combined with Dienogest on EMT patients and its effect on recurrence and pregnancy, in order to provide a new drug pathway for the treatment of this type of disease. The results showed that the clinical efficacy, rate of cumulative pregnancy and live birth in GD group were markedly above the DT group, and the recurrence rate, antibody positivity rate, sex hormone level, serum inflammation indicator, VAS score and the adverse reactions incidence in GD group were markedly below the DT group. No remarkable discrepancy in the rates of miscarriage, multiple pregnancies and ectopic pregnancies in both groups. It shows that the efficacy of GnRH-a combined with Dienogest in treatment of EMT is remarkable, which effectively improves the positive rate of antibody indicators and the level of sex hormones of the patients, improves the pregnancy situation, reduces the inflammatory reaction, and has a low recurrence rate, which provides a new refe-rence method for the clinical treatment of the related diseases. However, the present study has a small sample size and a short course of clinical medication, failing to observe the long-term effectiveness of this method of treatment. Due to the limitation of the conditions, more specific indices, such as others, could not be added. Multi-center, large-sample, high-quality clinical studies can be carried out in the later stage for validation.
